# Recent Advances in Understanding the Structures of Translesion Synthesis DNA Polymerases

**DOI:** 10.3390/genes13050915

**Published:** 2022-05-20

**Authors:** Justin A. Ling, Zach Frevert, M. Todd Washington

**Affiliations:** Department of Biochemistry, University of Iowa College of Medicine, Iowa City, IA 52242-1109, USA; justin-ling@uiowa.edu (J.A.L.); zach-frevert@uiowa.edu (Z.F.)

**Keywords:** DNA replication, DNA polymerases, DNA repair, DNA damage, mutagenesis, genome stability

## Abstract

DNA damage in the template strand causes replication forks to stall because replicative DNA polymerases are unable to efficiently incorporate nucleotides opposite template DNA lesions. To overcome these replication blocks, cells are equipped with multiple translesion synthesis polymerases that have evolved specifically to incorporate nucleotides opposite DNA lesions. Over the past two decades, X-ray crystallography has provided a wealth of information about the structures and mechanisms of translesion synthesis polymerases. This approach, however, has been limited to ground state structures of these polymerases bound to DNA and nucleotide substrates. Three recent methodological developments have extended our understanding of the structures and mechanisms of these polymerases. These include time-lapse X-ray crystallography, which allows one to identify novel reaction intermediates; full-ensemble hybrid methods, which allow one to examine the conformational flexibility of the intrinsically disordered regions of proteins; and cryo-electron microscopy, which allows one to determine the high-resolution structures of larger protein complexes. In this article, we will discuss how these three methodological developments have added to our understanding of the structures and mechanisms of translesion synthesis polymerases.

## 1. Introduction

DNA damage, which results from a variety of endogenous and exogenous sources, interferes with normal DNA replication and is a leading cause of genome instability. When the replication fork encounters DNA damage in the template strand, the replicative polymerases such as DNA polymerase delta (pol δ) and DNA polymerase epsilon (pol ε) stall. This is because these replicative polymerases are unable to efficiently incorporate nucleotides opposite the template DNA lesion. Thus, cells are equipped with multiple translesion synthesis (TLS) polymerases that have evolved specifically to incorporate nucleotides opposite DNA lesions [1,2,3,4,5,6,7,8,9,10,11,12]. While a variety of DNA polymerases are capable of carrying out TLS to a limited extent, five DNA polymerases appear to be the predominant TLS polymerases in eukaryotes. Among these are DNA polymerase eta (pol η), DNA polymerase iota (pol ι), DNA polymerase kappa (pol κ), Rev1, and DNA polymerase zeta (pol ζ). Pol η, pol ι, pol κ, and Rev1 are members of the Y-family of DNA polymerases, and pol ζ is a member of the B-family of DNA polymerases [13].

Depending on the type of DNA lesion at the stalled replication fork, TLS may require the catalytic function of either one TLS polymerase or two TLS polymerases [2]. In the case of TLS by one polymerase, the single TLS polymerase both inserts a nucleotide opposite the template lesion and extends from the resulting damaged primer–terminal base pair by incorporating subsequent nucleotides. In this capacity, the single TLS polymerase functions as both an “inserter” and an “extender”. An example of this is the bypass of 8-oxoguanine by pol η, which functions as both an inserter and an extender [14,15]. In the case of TLS by two polymerases, the first polymerase functions as an “inserter” by incorporating a nucleotide opposite the DNA lesion. The second polymerase functions as an “extender” by incorporating subsequent nucleotides. Only by the combined action of both polymerases can the DNA damage be bypassed. An example of this is the bypass of an abasic site by Rev1 (that functions as an inserter) and pol ζ (that functions as an extender) [16].

Each TLS polymerase that functions as an inserter has one or more cognate lesions opposite that it has evolved to incorporate nucleotides efficiently [1,6,7,11,17]. For example, the cognate lesions of pol η include thymine dimers and 8-oxoguanines [14,15,18,19,20]. The cognate lesions of pol ι include minor-groove purine adducts and exocyclic purine adducts [21,22]. The cognate lesions of pol κ include minor-groove adducts [23,24,25]. Finally, the cognate lesions of Rev1 include minor-groove guanine adducts and abasic sites [16,17,26,27].

X-ray crystallography has contributed enormously to our understanding of why certain types of DNA damage are cognate lesions for different polymerases. For example, X-ray crystallography has shown that pol η efficiently incorporates nucleotides opposite lesions such as thymine dimers and 8-oxoguanines by having a more spacious active site, which accommodates these geometrically distorted lesions [28,29,30,31]. This polymerase then utilizes Watson–Crick base pairing between the incoming nucleotide and the template base to direct nucleotide incorporation. X-ray crystallography has also shown that pol ι efficiently incorporates nucleotides opposite minor-groove purine adducts and exocyclic purine adducts by rotating the template purine base from the normal *anti* conformation to the *syn* conformation [22,32]. This polymerase then utilizes Hoogsteen base pairing between the incoming nucleotide and the template to direct incorporation. Crystallography has also shown that Rev1 efficiently incorporates nucleotides opposite minor-groove guanine adducts and abasic sites using a remarkable mechanism [33,34,35]. The template base is evicted from the double helix and an arginine side chain acts as the template for incorporation of dCTP.

While these past structural studies have provided a wealth of information about the mechanism of TLS polymerases, they have been limited to ground state structures of the polymerase domains. Three recent methodological developments have extended our understanding of the structures of these polymerases. These include time-lapse X-ray crystallography, which allows one to identify novel reaction intermediates; full-ensemble hybrid methods, which allow one to combine molecular simulations with small-angle X-ray scattering (SAXS) data; and cryo-electron microscopy (cryo-EM), which allows one to determine the high-resolution structures of large protein complexes. In this article, we will discuss how these three methodological developments have added to our understanding of the structures and mechanisms of TLS polymerases.

## 2. Time-Lapse X-ray Crystallography

While traditional X-ray crystallography has provided a wealth of information about the mechanisms of TLS polymerases, these structures have been limited to the protein alone, the binary complex formed by the protein and the DNA substrate, and the ternary complex formed by the protein, the DNA substrate, and the incoming dNTP substrate. What these studies have not been able to provide are structures of complexes formed by the protein, the DNA product, and the pyrophosphate product. These studies have also not provided the structures of any intermediates formed during the nucleotide-incorporation reaction. These limitations have been overcome recently by the use of time-lapse crystallography. 

In time-lapse crystallography, a ternary complex of the protein, the DNA substrate, and the incoming dNTP substrate are crystallized in the presence of Ca^2+^ [36,37,38,39]. The Ca^2+^ metal ions allow for the formation of this substrate ternary complex without allowing the reaction to proceed. Protein crystals are harvested and transferred to a solution containing Mg^2+^. The Mg^2+^ metal ions diffuse into the protein crystal and replace the Ca^2+^ ions thereby initiating the reaction. After various reaction times, often ranging from approximately 30 s to several minutes, the crystals are flash frozen in liquid nitrogen. X-ray diffraction data are then collected with the frozen crystals and the X-ray crystal structure is determined. This yields a series of structural snapshots at different stages of the nucleotide-incorporation reaction, and this has led to the discovery of several important and unexpected reaction intermediates. These new findings will be discussed below.

### 2.1. DNA Polymerase Eta

**Figure 1:**
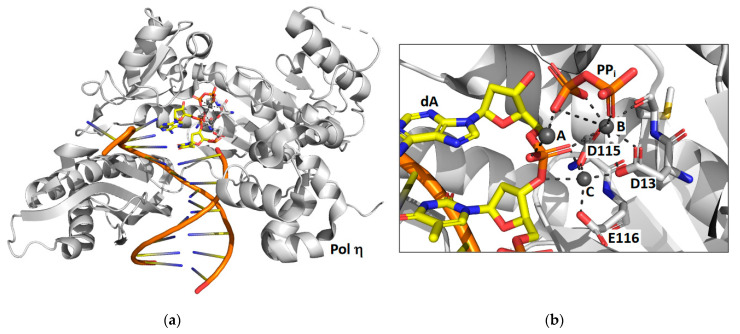
Time-lapse X-ray crystallography of the pol η catalytic core. Shown are the overall structure of the pol η product complex bound to DNA (**a**) and a close up of the active site with the positions of the pyrophosphate product (PP_i_), three Mg^2+^ ions (A, B, and C), and key amino acid residues (D13, D115, and E116) highlighted (**b**). For the products, carbon atoms are shown in yellow, nitrogen atoms are shown in blue, oxygen atoms are shown in red, and phosphorus atoms are shown in orange. This figure was generated with 4ECV.pdb [38].

Time-lapse X-ray crystallography was first applied to the catalytic core of human pol η [38] to observe the incorporation of an incoming dATP opposite a non-damaged thymine template (Figure 1). X-ray crystal structures were determined after various reaction times ranging from 40 to 300 s. The structure after 40 s of reaction time showed the ternary substrate complex of pol η, DNA, and dNTP with no evidence of product formation. The structure after 80 s of reaction time shows that some of the substrate has been converted to product. The structure after 230 s of reaction time shows that approximately 60% of the substrate has been converted to product and the other 40% remains substrate. Overall, the reaction proceeds 20 to 100-fold slower in the crystal than it does in solution. This is likely due to differences in the crystal and solution environments such as reduced thermal motions.

These structural snapshots show that the incoming nucleotide and the template base maintain Watson–Crick base pairing during the course of the reaction. Moreover, the overall global structure of the protein and the DNA does not change during the nucleotide incorporation reaction. However, notable local changes within the active site are visible. For example, the sugar pucker of the incoming nucleotide changes from 2′-endo in the substrate complex to 3′-endo in the product complex. This makes the minor groove shallower and causes the double-stranded region of the DNA substrate to adopt an A form conformation once the nucleotide has been incorporated.

The most striking feature observed during the formation of product is the appearance of a third Mg^2+^ ion that coordinates the non-bridging oxygens of the α- and β-phosphates. This is an important discovery because up to this time, polymerases were believed to utilize a two-metal ion mechanism. The presence of this third metal ion has directly challenged this view. The authors suggest that this third metal ion may stabilize the accumulating charge during the chemical step of phosphodiester bond formation and that the binding of the third metal ion occurs prior to and is necessary for the chemical step. It should be noted that a third metal ion was also found to be present in the active site of the DNA repair polymerase pol β [37].

Controversy about the role of this third metal ion intermediate persists in the field, because the kinetics of the appearance of this ion in the time-lapse crystallography is identical to the kinetics of phosphodiester bond formation. Thus, based on these structural studies alone, it is not possible to differentiate between the two mechanisms. In the first possible mechanism, the third metal ion binds first, which leads to an immediate phosphodiester bond formation. In such a mechanism, favored by the authors of the pol η study [38], the third metal ion would play an essential role in catalyzing nucleotide incorporation. In the second possible mechanism, the phosphodiester bond is formed first, which leads to an immediate third metal ion binding. In such a mechanism, supported by computational and crystallographic work with pol β [40], binding the third metal ion does not facilitate nucleotide incorporation. In fact, a recent comprehensive review of the literature has argued that the binding of the third metal ion prior to nucleotide incorporation actually inhibits the catalytic step [41]. More work is needed to resolve this important controversy in our mechanistic understanding of DNA polymerases.

### 2.2. Rev1

**Figure 2:**
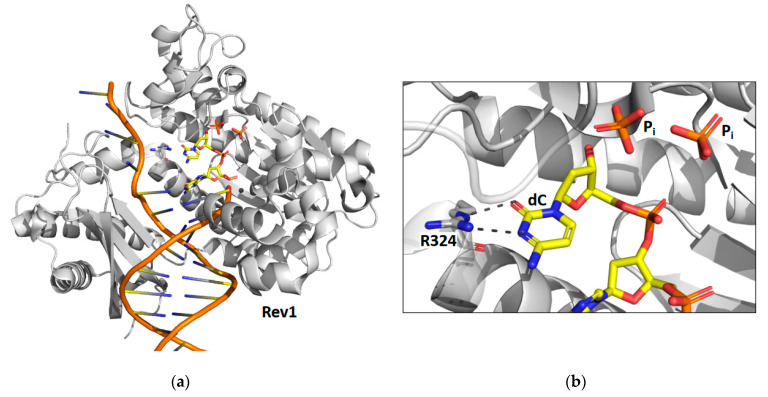
Time-lapse X-ray crystallography of the Rev1 catalytic core. Shown are the overall structure of the Rev1 product complex bound to DNA (**a**) and a close-up of the active site with the positions of the two inorganic phosphate products (P_i_) and a key amino acid residue (R324) highlighted (**b**). For the products, carbon atoms are shown in yellow, nitrogen atoms are shown in blue, oxygen atoms are shown in red, and phosphorus atoms are shown in orange. This figure was generated with 6X72.pdb [39].

Time-lapse X-ray crystallography was recently applied to the catalytic core of yeast Rev1 [39] to observe the incorporation of an incoming dCTP opposite a non-damaged guanine template (Figure 2). X-ray crystal structures were determined after various reaction times ranging from 5 to 30 min. The structure after 5 min of reaction time shows that approximately 30% of the substrate has been converted to product and the other 70% remains as substrate. The structure after the 10 min reaction time shows that all of the substrate has been converted to product. Like pol η, the reaction proceeds significantly slower in the crystal than it does in solution. Unlike pol η, the structural snapshots of Rev1 provided no evidence for the existence of a third metal ion.

The structural snapshots of Rev1 show that the guanine template remains extrahelical and binds within the template-binding pocket in the active site during the entire nucleotide-incorporation reaction. It does not leave this pocket after the reaction is complete. In the structure of the substrate complex, the incoming dNTP binds to the sidechain of arginine-324. The atoms of the guanidino group of the arginine side chain and the atoms of the cytosine ring are all within the same plane allowing for the formation of two hydrogen bonds between the arginine side chain and the cytosine base. In the structure of the product complex, the incorporated cytosine shifts nearly 2 Å toward its neighboring base in the primer strand. This results in the loss of planarity between the arginine side chain and the cytosine base and a weakening of the two hydrogen bonds between them.

The most striking feature observed during the nucleotide-incorporation reaction is the presence of two inorganic phosphates in the active site instead of the usual the pyrophosphate product. These unexpected phosphate intermediates are not associated with any metal ions. The presence of these phosphate ions imply that Rev1 possesses a novel pyrophosphatase activity, which cleaves the pyrophosphate product into two phosphates immediately after nucleotide incorporation. Presumably this activity thermodynamically drives the nucleotide-incorporation reaction forward by the removal of the pyrophosphate product. Moreover, the production of inorganic phosphate by Rev1 was also shown to occur during nucleotide incorporation in solution.

X-ray crystal structures of Rev1 were determined after 15 and 30 min to better understand the fate of the two phosphate ion products. The structure after the 15 min reaction time showed that the β phosphate was released while the γ phosphate remained bound in the active site. The structure after the 30 min reaction time showed that the γ phosphate was also released. These structures show an ordered mechanism of phosphate ion release with the β phosphate releasing before the γ phosphate. Several unresolved issues remain regarding the mechanism of Rev1’s novel pyrophosphatase activity. For example, the identity of the general base that activates water for a nucleophilic attack on the pyrophosphate has yet to be determined. In addition, is the pyrophosphatase reaction necessary for efficient nucleotide incorporation? More work is needed to address these important questions.

## 3. Full-Ensemble Hybrid Methods

SAXS is an experimental technique in which one places a solution of a protein in an X-ray beam and measures the continuous pattern of scattered X-rays as a function of distance from the center of the detector. The analysis of this scattering data, which have been described elsewhere [42,43,44,45,46,47,48,49], allows one to determine the radius of gyration of the protein (R_g_), the maximal distance between pairs of atoms in the protein (D_max_), and the distribution of distances between every pairwise combination of atoms in the protein. This latter information is depicted in the pairwise (P(r)) distribution plot. In the case of highly structured proteins with little conformational flexibility, the information in the P(r) plot can be used for ab initio shape predictions, which provide the overall shape of the protein at a medium to low resolution (about 10 to 20 Å).

In the case of proteins with a large amount of intrinsic disorder or a high degree of conformational flexibility, SAXS data are typically analyzed by carrying out a minimal ensemble search. A common strategy for performing minimal ensemble searches is BILBOMD [50]. This entails running a molecular dynamics simulation where the conformations of individual domains are largely modeled as rigid bodies with flexible regions between them, allowing these domains to adopt a variety of orientations relative to one another. These molecular dynamics simulations typically utilize timesteps of 1 to 2 fs with a structure recorded every 0.5 ps. Thus, a 5 ns-simulation will yield a pool of about 10,000 structures. BILBOMD then uses a genetic algorithm to find the minimum ensemble (usually a weighted set of 2 to 5 structures from the larger pool) that best fits the data. By seeking the minimal ensemble that best accounts for the experimental SAXS data, one can attempt to avoid the overfitting problem that is often encountered in SAXS analyses. 

The primary drawback of minimal-ensemble methods, which represent conformationally flexible proteins using a weighted set of only 2 to 5 static structures, is that it results in highly unrealistic models of such proteins. The vast number of the intermediate states between these few static structures are not represented in the final ensemble. Recently, a full-ensemble method has been developed to overcome this problem [49,51]. By using coarse-grained Langevin dynamics simulations, one can generate a full ensemble of tens of thousands of structures. Instead of searching for a minimal ensemble that best fits the experimental data, the entire ensemble is directly compared to the SAXS experimental data. The R_g_ and D_max_ values obtained from the entire ensemble are directly compared to the experimentally determined R_g_ and D_max_ values. Moreover, the P(r) curve obtained from the full ensemble is directly compared to the experimentally determined P(r) curve. This allows one to overcome the overfitting problem by not relying on curve fitting at all. In addition, this method, which represents conformationally flexible proteins by an ensemble of over ten thousand structures, results in more realistic models of highly flexible proteins. 

**Figure 3:**
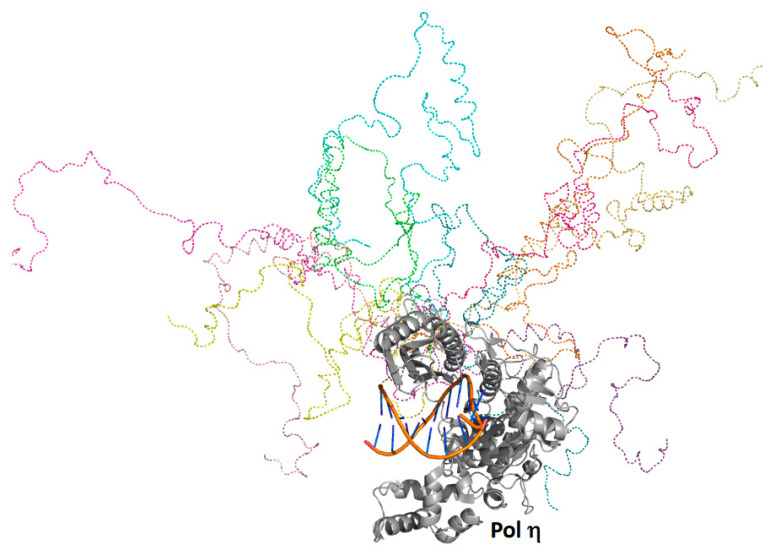
Full-ensemble hybrid methods with full-length pol η. Shown is an overlay of ten randomly selected structures from the full ensemble generated for pol η as described [51]. The catalytic core and the DNA of these ten structures are superimposed, and the intrinsically disordered C-terminal regions of these ten structures are depicted in different colors as dashed lines.

Yeast pol η was predicted to possess a folded catalytic core (residues 1 to 510) as well as a C-terminal intrinsically ordered region (residues 510 to 632), which contains several α-helicases and a small, folded ubiquitin-binding, zinc-binding (UBZ) motif (residues 550 to 580) [52] and two distinct proliferating cell nuclear antigen (PCNA)-interacting protein (PIP) motifs [53]. Full-ensemble hybrid methods were used to test this prediction and to assess the conformational flexibility of the C-terminal region of pol η [51] (Figure 3). Langevin dynamics simulations were performed using a coarse-grained model of full-length pol η, whereby each amino acid residue was represented by one to four coarse-grained beads. Timesteps of 125 fs were used, and a structure was recorded every 1 ns over the duration of a 10-μs simulation generating an ensemble of 10,000 structures. R_g_ and D_max_ values were obtained for the entire ensemble, and these theoretical values were directly compared to those obtained using SAXS. The theoretical and experimental R_g_ and D_max_ values and the theoretical and experimental P(r) curves were in close agreement.

Analysis of this experimentally validated ensemble showed that the C-terminal region of pol η is indeed mostly unstructured and samples a wide range of conformational space. Approximately 5% of the time, the C-terminal region is compact and remains near the catalytic core. In another approximate 5% of the time, the C-terminal region is highly extended, stretching far from the catalytic core. The remaining 90% of the time, the C-terminal region occupies a wide range of conformations in between these two extremes. These findings support a model in which the C-terminal region acts as a flexible tether linking the catalytic core of pol η to ubiquitin-modified PCNA, a core component of bypass complexes. As pol ι, pol κ, and Rev1 also possess similar disordered C-terminal regions [10,54], this tethering likely facilitates polymerase sampling, which would be needed to ensure that the most appropriate TLS polymerase is selected to bypass a given type of DNA damage in accordance with the kinetic selection model, which is described elsewhere [11]. 

## 4. Cryo-Electron Microscopy

In recent years, cryo-EM has emerged as a major method for obtaining high-resolution structural information of large protein complexes. Compared to X-ray crystallography, cryo-EM requires small amounts of purified protein. These complexes are frozen in a thin layer of ice, and cryo-EM images are taken. Images of particles are averaged and 2D class averages are obtained. Ultimately, a 3D reconstitution of the electron density of the complex is determined. Two cryo-EM studies of TLS polymerases have led to significant advances in our understanding of the mechanisms of these proteins. These two studies will be discussed below. 

### 4.1. DNA Polymerase Zeta

In many cases, TLS requires the catalytic function of two polymerases [2]. The first polymerase functions as an “inserter” to catalyze the incorporation of a nucleotide opposite the template lesion, and the second polymerase functions as an “extender” to catalyze the incorporation of an additional nucleotide following the resultant primer–terminal base pair with the lesion in the template strand. Pol ζ functions as an “extender” during the bypass of a wide range of DNA lesions [16,55]. Pol ζ is a member of the B-family of DNA polymerases along with replicative polymerases pol α, pol δ, and pol ε [13]. Yeast pol ζ was initially believed to be comprised of four subunits: the Rev3 catalytic subunit and the Rev7, Pol31, and Pol32 non-catalytic subunits [56,57,58]. The Rev3 subunit is large (173 kDa), while the Rev7, Pol31, and Pol32 subunits are small (29 kDa, 55 kDa, and 40 kDa, respectively). Interestingly, the Pol31 and Pol32 subunits are also subunits of replicative pol δ. Attempts to determine the X-ray crystal structure of the entire pol ζ protein have been unsuccessful in part due to the difficulty of obtaining sufficient quantities of the protein for crystallization. Instead, X-ray crystal structures have only been determined for small portions of pol ζ, such as the Rev7 non-catalytic subunit bound to a peptide containing 28 amino acid residues of the Rev3 catalytic subunit [59].

**Figure 4:**
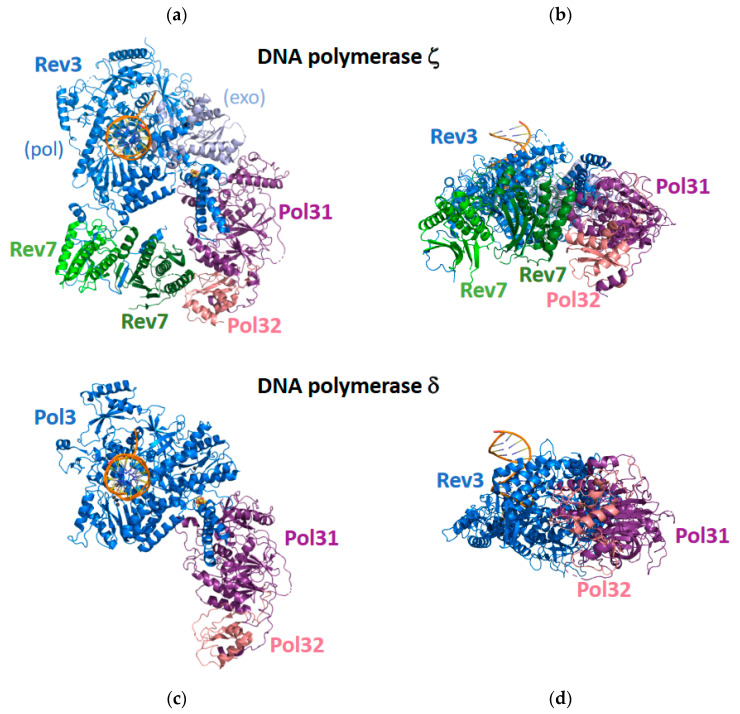
Cryo-EM structure of the full pol ζ complex. Shown are a top view (**a**) and a side view (**b**) of pol ζ. The polymerase domain of the Rev3 subunit is shown in blue, and the exonuclease domain of the Rev3 subunit is shown in light blue. The two Rev7 subunits are shown in green and light green. The Pol31 subunit is shown in purple, and the Pol32 subunit is shown in pink. Shown are a top view (**c**) and a side view (**d**) of pol δ. The catalytic Pol3 subunit is shown in blue, the Pol31 subunit is shown in purple, and the Pol32 subunit is shown in pink. This figure was generated with 7S0T.pdb [60] and 6P1H.pdb [61].

Cryo-EM was recently used to determine the structures of the entire yeast pol ζ protein alone and bound to a DNA substrate and an incoming dNTP [60] (Figure 4). The most surprising feature of these structures is that pol ζ contains two Rev7 subunits, denoted Rev7_A_ and Rev7_B_, rather than the expected one Rev7 subunit. These five subunits—Rev3, Rev7_A_, Rev7_B_, Pol31, and Pol32—form a pentameric ring-like structure. The two Rev7 subunits bind to an extended, disordered region of the Rev3 subunit, and the DNA substrate binds exclusively to the Rev3 catalytic subunit.

The overall structure of pol ζ resembles the structure of pol δ, which was also determined by cryo-EM [61] (Figure 4). In the case of pol δ, the catalytic Pol3 subunits bind the DNA substrate. The non-catalytic Pol31 subunit binds to Pol3 and to the non-catalytic Pol32 subunit, but Pol 3 and Pol32 do not directly interact with each other. In the case of pol ζ, the catalytic Rev3 subunit binds the DNA substrate. The Pol31 subunit binds to Rev3 and to the Pol32 subunit, but Rev3 and Pol32 do not directly interact with each other. The Rev7_A_ subunit binds the extended loop of the Rev3 subunit and binds the Rev7_B_ subunit. The Rev7_B_ subunit binds the extended loop of the Rev3 subunit and binds the Rev7_A_ and Pol32 subunit, thereby closing the ring-like structure. 

The structure of the catalytic Rev3 subunit of pol ζ resembles the structure of the Pol3 subunit of pol δ. These subunits have two domains: an N-terminal exonuclease domain and a C-terminal polymerase domain. The C-terminal polymerase domains contain three subdomains found in most DNA polymerases: palm, fingers, and thumb. In both pol ζ and pol δ, the palm subdomain contains several conserved acid amino acid residues that coordinate the metal ions necessary for catalyzing the nucleotide incorporation reaction. In both proteins, the fingers subdomain contacts the nascent base-pair ensuring that nucleotide incorporation occurs with high fidelity. In the case of pol δ, the N-terminal exonuclease domain contains conserved acid amino acids that coordinate the catalytically essential metal ions. In the case of pol ζ, the N-terminal exonuclease domain is not active, which contributes to its ability to carry out the extension step of TLS.

The most important difference observed between the structures of the catalytic domains of pol ζ and pol δ is the conformation of the extended loop connecting the N-terminal exonuclease domains and the C-terminal polymerase domains. In the case of pol δ, this loop contacts the primer–terminal base pair. These contacts are likely the structural basis for the inability of pol δ to extend from aberrant primer–terminal base pairs containing mismatches or template DNA lesions. In the case of pol ζ, this loop is in a different conformation and does not contact the primer–terminal base pair. This lack of contacts is likely the structural basis for the remarkable ability of pol ζ to extend from primer–terminal base pairs containing mismatches or template lesions. Thus, it is likely that this structural difference between these two proteins is what allows pol ζ to fulfill its role as an “extender” during TLS.

### 4.2. DNA Polymerase Kappa and PCNA

Pol κ has two functions in TLS. First, it functions as an “inserter” to incorporate nucleotides opposite minor-groove purine adducts [23,24,25]. Second, it functions as an “extender” during the bypass of a wide range of DNA lesions [62,63,64]. Pol κ is a member of the Y-family of DNA polymerases. Multiple X-ray crystal structures of human pol κ have been solved [64,65,66]. These structures show that the polymerase domain has the usual fingers, thumb, and palms subdomains. Like pol η, the incoming nucleotide and the templating base form Watson–Crick base pairs. Unlike pol η, however, the active site of pol κ is too small to accommodate geometrically distorting DNA lesions. Instead, there is a pocket between the fingers and little fingers subdomains into which minor groove adducts can be accommodated [66]. This is likely the structural basis for pol κ functioning as an “inserter”. In addition, pol κ contains a region called the “N clasp”, which is not found in other Y-family polymerases. The “N clasp” allows pol κ to completely encircle the DNA substrate, which may increase the processivity of pol κ on aberrant primer termini thereby providing at least a partial structural explanation of its “extender” function [64].

**Figure 5:**
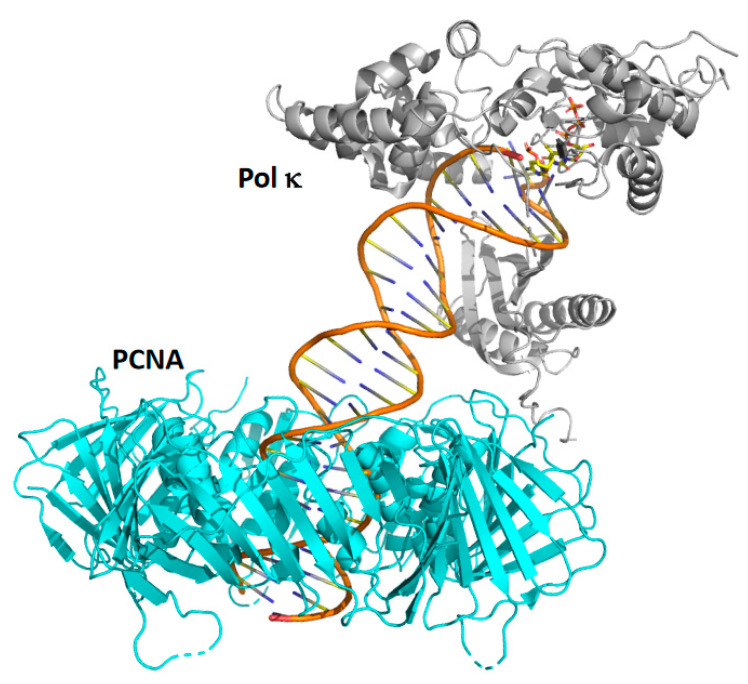
Cryo-EM structure of full-length pol κ bound to PCNA and DNA. Shown is a side view of pol κ bound to PCNA and DNA. Pol κ is shown in grey, and PCNA is shown in cyan. For the substrates in the active site, carbon atoms are shown in yellow, nitrogen atoms are shown in blue, oxygen atoms are shown in red, and phosphorus atoms are shown in orange. This figure was generated with 7NV0.pdb [67].

While X-ray crystallography has focused on the polymerase domain of pol κ, little effort has been devoted to studies of the C-terminal unstructured region of the protein, which is approximately 300 amino acid residues in length. This unstructured region contains motifs for binding the trimeric DNA sliding clamp PCNA, ubiquitin, and Rev1 [10,54]. Recently, the cryo-EM structures of full-length pol κ bound to DNA and PCNA and to DNA and ubiquitin-modified PCNA (Ub-PCNA), a post-translationally modified form of PCNA that functions in TLS, have been determined [67] (Figure 5). These structures both show that pol κ binds to the DNA substrate on the front face of the PCNA ring. In the structure of pol κ bound to DNA and unmodified PCNA, the C-terminal unstructured region of pol κ is not visible, except for the PIP motif, an eight amino acid motif that binds within a hydrophobic pocket on the front face of the PCNA ring. 

In the structure of pol κ bound to DNA and Ub-PCNA, little electron density is visible for the ubiquitin moieties attached to the two PCNA subunits that are not directly binding the pol κ. This suggests that these ubiquitin moieties are flexible and can occupy a large number of conformations. The ubiquitin moiety attached to the PCNA subunit that directly binds pol κ appears to have a stronger density. This suggests that pol κ limits the conformational flexibility of this ubiquitin moiety. Interestingly there is no electron density for the UBZ motif of pol κ, which supposedly binds the ubiquitin moiety on Ub-PCNA. The authors suggest the lack of density for the UBZ motif is likely due to the transient nature of the interaction between the UBZ and ubiquitin. 

## 5. Conclusions

In this review, we discussed several recent approaches to improving our understanding of the structures and mechanisms of TLS polymerases. Time-lapse X-ray crystallography has led to the identification of new features of the nucleotide incorporation reaction, including the discovery of a third metal ion in pol η [38] and a novel pyrophosphatase activity in Rev1 [39]. Full-ensemble hybrid methods have led to better description of the conformational flexibility in the intrinsically disordered region of pol η [51]. Cryo-EM has led to the determination of structures of pol ζ [60] and the complex of pol κ on DNA and PCNA [67]. The use of these new approaches is in its infancy, and much more structural and mechanistic information will undoubtably be forthcoming in the years ahead.

In addition to these studies of TLS polymerases, similar studies are being carried out with classical DNA polymerases—those involved in normal DNA replication and repair. For example, time-lapse crystallography has been applied to pol β, a classical polymerase involved in base excision repair [36,37]. Similarly, cryo-EM has been used to determine the structure of pol δ [61] and pol ε [68]. Carefully comparing and contrasting the structures and mechanisms of classical polymerases and TLS polymerases will lead to an integrated understanding of how DNA polymerases synthesize DNA—a process necessary for the maintenance of genome stability.

## Abbreviations

Cryo-EM: cryo-electron microscopy; D_max_: maximum distance; PCNA: proliferating cell nuclear antigen; PIP: PCNA-interacting protein; Pol δ: DNA polymerase delta; Pol ε: DNA polymerase epsilon; Pol η: DNA polymerase eta; Pol ι: DNA polymerase iota; Pol κ: DNA polymerase kappa; Pol ζ: DNA polymerase zeta; P(r): pairwise distribution; R_g_: radius of gyration; SAXS: small-angle X-ray scattering; TLS: translesion synthesis; Ub-PCNA: ubiquitin-modified PCNA; UBZ: ubiquitin-binding/zinc-binding.
